# Supplementary material for: PhyloMagnet: fast and accurate screening of short-read meta-omics data using gene-centric phylogenetics
Source: Bioinformatics. 2019 Oct 24;36(6):1718–24. doi: 10.1093/bioinformatics/btz799 (PMC7703773; doi:10.1093/bioinformatics/btz799)
Supplement: btz799_Supplementary_Data [file btz799_supplementary_data.pdf]

Contents

Download and prepare Reference sequences **1**
Prepare initial rp16 references . . . 1
Create the extended rp16 references including all genera included in the MBARC-26 dataset . . . 2
Get and prepare Chloroplast gene references from uniprot. . . . 3

Download and prepare query datasets **4**
MBARC-26 . . . 5
Tara Oceans . . . 5
Coral Bleaching . . . 5

Download published results as comparison for benchmarking **6**
MBARC-26 . . . 6
Tara Oceans . . . 6
Coral Bleaching . . . 6

Run Benchmarks **8**
MBARC-26 benchmark . . . 8
PhyloMagnet . . . 8
GraftM . . . 8
Leave one out benchmark . . . 9
Kraken2 . . . 13
Tara Southern Oceans Benchmark . . . 14
Coral Bleaching Benchmark . . . 14

Evaluate Benchmarks **14**
Fig 2: . . . 14
Fig 3: . . . 17
Fig 4: . . . 17
Fig S1: . . . 17
Fig S2: . . . 20
Fig S3: . . . 20
Fig S4: . . . 21
Table S1: . . . 25
Table S2: . . . 26

References **28**

Download and prepare Reference sequences

Prepare initial rp16 references

Download initial references from EggNOG and create multiple sequence alignments and trees. Save the reference packages in compressed form in `rp16_rpkg`.

```
singularity pull --name PhyloMagnet.simg shub://maxemil/PhyloMagnet:latest
singularity exec PhyloMagnet.simg python3 -c "import ete3; ncbi = ete3.NCBITaxa()"

nextflow run maxemil/PhyloMagnet \
            -with-singularity  PhyloMagnet.simg \
            --align_method 'mafft-einsi' \
            --phylo_method 'iqtree' \
```

```
            --cpus 36 \
            --megan_vmoptions "../MEGAN.vmoptions" \
            --reference_classes MBARC/eggnog_rp16.txt
            --reference_dir rp16_references

bash $HOME/.nextflow/assets/maxemil/PhyloMagnet/utils/make_reference_packages.sh \
            rp16_references rp16_rpkg
```

Create HMM models for the alignments of reference sequences to search additional genomes and complement the reference OGs.

```
mkdir rp16_hmms

for cog in rp16_references/C*/COG*[0-9].unique.aln;
do
  singularity exec -B $PWD:$PWD benchmark.simg hmmbuild rp16_hmms/$(basename
      ${cog%%.unique.aln}).hmm $cog;
done
```

Create the extended rp16 references including all genera included in the MBARC-26 dataset

Download the genomes of relatives of the MBARC species, annotate each sequence with the taxid

```
download(){
  while read id tax;
  do
    ass=$(wget -q
        "https://www.ncbi.nlm.nih.gov/entrez/eutils/esearch.fcgi?db=assembly&term="$id
        -O - | xml_grep -cond Id --text_only)
    ftp=$(wget -q
        "https://www.ncbi.nlm.nih.gov/entrez/eutils/esummary.fcgi?db=assembly&id="$ass
        -O - | xml_grep -cond FtpPath_GenBank --text_only)
    base=$(basename $ftp)
    wget -q $ftp/$base"_protein.faa.gz" -O $id.faa.gz
    wget -q $ftp/$base"_genomic.fna.gz" -O $id.fna.gz
    zcat "$f.faa.gz" | sed 's/>/>'"$tax"'\./g' | gzip > "$f.labelled.faa.gz" ;
  done < $1

  zcat *.labelled.faa.gz | gzip > all_proteomes.faa.gz
}

cd MBARC/genomes_relatives
download taxids.txt
cd ../..
```

Use the HMM to search for sequences in the relatives' genomes and add these sequences to the reference fasta

```
mkdir rp16_added_fasta

for hmm in rp16_hmms/COG*[0-9].hmm;
do
```

```
  singularity exec -B $PWD:$PWD benchmark.simg hmmsearch -T 50 --tblout rp16_added_fasta/$(basename ${hmm%%.hmm}).out $hmm MBARC/genomes_relatives/all_proteomes.faa.gz;
  grep -v "^#" rp16_added_fasta/$(basename ${hmm%%.hmm}).out | awk '{print $1}' | esl-sfetch -f MBARC/genomes_relatives/all_proteomes.faa.gz - > rp16_added_fasta/$(basename ${hmm%%.hmm}).hits.fasta ;
done

for cog in  rp16_references/C*/COG*[0-9].fasta;
do
  cat $cog rp16_added_fasta/$(basename ${cog%%.fasta}).hits.fasta > rp16_added_fasta/$(basename ${cog%%.fasta})_add.fasta
done
```

Use PhyloMagnet to create alignments and trees for each of the extended reference set. Package the files into reference packages (rpkg).

```
nextflow run maxemil/PhyloMagnet \
            -with-singularity PhyloMagnet.simg \
            --align_method 'mafft-einsi' \
            --phylo_method 'iqtree' \
            --cpus 36 \
            --megan_vmoptions "../MEGAN.vmoptions" \
            --local_ref "rp16_added_fasta/*_add.fasta" \
            --reference_dir rp16_added_references -resume

bash $HOME/.nextflow/assets/maxemil/PhyloMagnet/utils/make_reference_packages.sh rp16_added_references rp16_added_rpkg
```

Analogous to the rpkgs, create gpkgs to be used with graftM

```
mkdir rp16_added_gpkg

for cog in rp16_added_references/COG*;
do
  base=$(basename $cog)
  singularity exec -B $PWD:$PWD GraftM/graftM.img graftM create \
            --sequences $cog/$base.unique.fasta \
            --alignment $cog/$base.unique.aln \
            --rerooted_tree $cog/$base.treefile \
            --taxonomy $cog/$base.taxid.map \
            --output rp16_added_gpkg/$base.gpkg
done
```

Get and prepare Chloroplast gene references from uniprot.

Print the FASTA header in the format TAXID.ACCESSION, similar to how EggNOG references are formatted.

```
clean_headers(){
  python3 <<<"""
from Bio import SeqIO
import re

```

```
pattern = re.compile('OX=[0-9]*')

for gene in ['atpA', 'atpB', 'petB', 'petD', 'psaA', 'psaB', 'psbA', 'psbB', 'psbC', 'psbD', 'psbE', 'psbI']:
    recs = []
    for rec in SeqIO.parse('{}.fasta'.format(gene),'fasta'):
        seqid = rec.id.split('|')[1]
        seqtax = pattern.search(rec.description).group(0).split('=')[1]
        rec.id = '{}.{}'.format(seqtax, seqid)
        rec.description = ''
        recs.append(rec)
    with open('{}.fasta'.format(gene), 'w') as outhandle:
        SeqIO.write(recs, outhandle, 'fasta')
  """
}

mkdir chloroplast_references_uniprot
cd chloroplast_references_uniprot

for gene in "atpA" "atpB" "petB" "petD" "psaA" "psaB" "psbA" "psbB" "psbC" "psbD" "psbE" "psbI";
do
  wget "https://www.uniprot.org/uniprot/?query=gene%3A\
  $gene+(reviewed%3Ayes+OR+dinophyceae)+(chloroplast+OR+plastid)&format=fasta" \
-O "$gene.fasta"
done
clean_headers
cd ..
```

Reconstruct alignmnents and trees for chloroplast genes and package them into rpkgs

```
nextflow run maxemil/PhyloMagnet \
            -with-singularity PhyloMagnet.simg \
            --cpus 40 \
            --local_ref "chloroplast_references_uniprot/*.fasta" \
            --megan_vmoptions "../MEGAN.vmoptions" \
            --phylo_method 'iqtree' \
            --align_method 'mafft-einsi' \
            --reference_dir 'chloroplast_references'

bash $HOME/.nextflow/assets/maxemil/PhyloMagnet/utils/make_reference_packages.sh \
            chloroplast_references/ chloroplast_rpkgs/
```

Download and prepare query datasets

Python script to download sra files from ENA and subsequently convert them to FASTQ files (basically taken from the template used in PhyloMagnet)

```
donwnload_fastq(){
  python3 <<<"""
import requests
import shutil
```

```
import subprocess

acc = '$1'
url = ''
if len(acc) == 9:
    url = 'http://ftp.sra.ebi.ac.uk/vol1/{}/{}/{}'.format(acc[0:3].lower(), acc[0:6],
        acc)
elif len(acc) == 10:
    url = 'http://ftp.sra.ebi.ac.uk/vol1/{}/{}/{}/{}'.format(acc[0:3].lower(), acc[0:6],
        "00" + acc[-1], acc)
elif len(acc) == 11:
    url = 'http://ftp.sra.ebi.ac.uk/vol1/{}/{}/{}/{}'.format(acc[0:3].lower(), acc[0:6],
        "0" + acc[-2:], acc)

r = requests.get(url, stream=True)
with open('$1.sra', 'wb') as f:
      shutil.copyfileobj(r.raw, f)
  """
  fastq-dump --gzip --readids --split-spot --skip-technical --clip $1.sra
}
```

MBARC-26

Download MBARC-26 Illumina metagenomic dataset from ENA

```
mkdir MBARC/fastq
cd MBARC/fastq
donwnload_fastq SRR3656745
```

Subsample 1% and 10% from the MBARC-26 data for benchmarking

```
seqtk sample -s11 <(gunzip -c SRR3656745.fastq.gz) 0.01 | pigz -9 >
    SRR3656745.1perc.fastq.gz
seqtk sample -s11 <(gunzip -c SRR3656745.fastq.gz) 0.1 | pigz -9 >
    SRR3656745.10perc.fastq.gz
```

Tara Oceans

Download Tara Oceans metagenomic data from ENA

```
mkdir Tara_Southern_Ocean/fastq
cd Tara_Southern_Ocean/fastq

for id in ERR598945 ERR599008 ERR599059 ERR599090 ERR599104 ERR599121 ERR599125
    ERR599176;do
  donwnload_fastq $id
done
```

Coral Bleaching

Download Coral Bleaching metatranscriptomic data from ENA, concatenate them to a single dataset.

```
mkdir Tara_Southern_Ocean/fastq
cd Tara_Southern_Ocean/fastq

for id in SRR5453739 SRR5453740 SRR5453741 SRR5453742 SRR5453743 SRR5453744 SRR5453745
    SRR5453746 SRR5453747 SRR5453748 SRR5453749 SRR5453750 SRR5453751 SRR5453752
    SRR5453753 SRR5453754 SRR5453755 SRR5453756 SRR5453757 SRR5453758 SRR5453759
    SRR5453760 SRR5453761 SRR5453762 SRR5453763 SRR5453764 SRR5453765;do
  donwnload_fastq $id
done
cat SRR*.fastq.gz > PRJNA377366.fastq.gz
```

Download published results as comparison for benchmarking

MBARC-26

Compare PhyloMagnet results to GraftM results, see file `MBARC/genomes_mapped.txt` for genome mapping information (i.e. abundance).

Tara Oceans

```
cd Tara_Southern_Ocean
wget https://ndownloader.figshare.com/files/8243654 -O NON_REDUNDANT_MAGs.tar.gz
tar -xzf NON_REDUNDANT_MAGs.tar.gz
# similarly, download raw BINs
```

Compute taxonomic labels for additional genome bins using sourmash (download the database from sourmash's website https://sourmash.readthedocs.io/en/latest/databases.html)q

```
cd sourmash
wget https://osf.io/nemkw/download -O t-genbank-k51.lca.json.gz
nextflow run sourmash.nf --genomes ../NON_REDUNDANT_MAGs/* --reference
    genbank-k51.lca.json.gz --outdir signatures-MAGS -qs 35

cd ..
```

Coral Bleaching

```
mkdir Coral_Bleaching/transcriptome
cd Coral_Bleaching/transcriptome

split_alignment(){
  python3 <<<"""
from Bio import SeqIO
refs = [rec.id for rec in SeqIO.parse('$2$1.unique.aln', 'fasta')]
queries = [rec for rec in SeqIO.parse('$1.refquer.aln', 'fasta') if not rec.id in refs]
with open('$1.quer.aln', 'w') as out:
    for rec in queries:
        SeqIO.write(rec, out, 'fasta')
"""
}
```

Download the metatranscriptomic assembly from GEO database. prepare blast database

```
TRANSCRIPTOME_FILE=GSE97888%5FMetatranscriptome%5Fqualityfiltered%2Efasta%2Egz
wget
    https://www.ncbi.nlm.nih.gov/geo/download/?acc=GSE97888&format=file&file=$TRANSCRIPTOME_FILE
    -O GSE97888.fasta.gz
gunzip GSE97888.fasta.gz
makeblastdb -in GSE97888.fasta -out GSE97888.db -dbtype nucl
```

For each reference OG, identify homologous transcripts and place them onto the ref tree.

```
cd ..
mkdir place_transcripts
cd place_transcripts

for dir in ../references/*/;
do
  gene=${dir#../references/}
  gene=${gene%/}
  tblastn -db ../transcriptome/GSE97888.db -num_threads 30 -query $dir$gene.unique.fasta
      -evalue 1e-15 -outfmt '6 qseqid sseqid pident length mismatch gapopen qstart qend
      sstart send sframe evalue bitscore' -out $gene.tblastn
  python3 ../get_blast_hsp.py -b $gene.tblastn -f ../transcriptome/GSE97888.fasta -o
      $gene.hits --translate -n qseqid sseqid pident length mismatch gapopen qstart qend
      sstart send sframe evalue bitscore
  python3 ../get_blast_hsp.py -b $gene.tblastn -f ../transcriptome/GSE97888.fasta -o
      $gene.nucl.hits -n qseqid sseqid pident length mismatch gapopen qstart qend sstart
      send sframe evalue bitscore
  singularity exec -B /local:/local ../../PhyloMagnet.simg trimal -in
      $dir$gene.unique.aln -out $gene.ref.phy -phylip
  singularity exec -B /local:/local ../../PhyloMagnet.simg papara -t $dir$gene.treefile
      -s $gene.ref.phy -q $gene.hits -a -n $gene -r
  singularity exec -B /local:/local ../../PhyloMagnet.simg epa-ng --split $gene.ref.phy
      papara_alignment.$gene
  mv query.fasta $gene.quer.aln
  singularity exec -B /local:/local ../../PhyloMagnet.simg epa-ng --ref-msa
      $dir$gene.unique.aln --tree $dir$gene.treefile --query $gene.quer.aln --model
      $dir$gene.modelfile --no-heur --threads 10
  mv epa_result.jplace $gene.jplace
  mv epa_info.log $gene.epa_info.log
  singularity exec -B /local:/local ../../PhyloMagnet.simg gappa analyze assign
      --jplace-path $gene.jplace --taxon-file $dir$gene.taxid.map --threads 10
  mv profile.csv $gene.csv
  rm labelled_tree
  mv per_pquery_assign $gene.assign
  singularity exec -B /local:/local ../../PhyloMagnet.simg gappa analyze graft
      --name-prefix 'Q_' --jplace-path $gene.jplace --threads 10
done
cd ../..
```

Run Benchmarks

MBARC-26 benchmark

PhyloMagnet

Run and time PhyloMagnet on MBARC data (full, 10% and 1% subsampled)

```
/usr/bin/time --output=queries_1perc_timed.txt -v nextflow run maxemil/PhyloMagnet \
            --lineage "genus" \
            -with-singularity  ../PhyloMagnet.simg \
            --cpus 10 \
            --reference_packages "../rp16_added_rpkg/*.tgz*" \
            --fastq "fastq/SRR3656745.1perc.fastq.gz" \
            --megan_vmoptions "../MEGAN.vmoptions" \
            -with-report MBARC_report_1perc.html \
            --queries_dir queries_1perc

/usr/bin/time --output=queries_10perc_timed.txt -v nextflow run maxemil/PhyloMagnet \
            --lineage "genus" \
            -with-singularity  ../PhyloMagnet.simg \
            --cpus 10 \
            --reference_packages "../rp16_added_rpkg/*.tgz*" \
            --fastq "fastq/SRR3656745.10perc.fastq.gz" \
            --megan_vmoptions "../MEGAN.vmoptions" \
            -with-report MBARC_report_10perc.html \
            --queries_dir queries_10perc

/usr/bin/time --output=queries_timed.txt -v nextflow run maxemil/PhyloMagnet \
            --lineage "genus" \
            -with-singularity  ../PhyloMagnet.simg \
            --cpus 10 \
            --reference_packages "../rp16_added_rpkg/*.tgz*" \
            --fastq "fastq/SRR3656745.fastq.gz" \
            --megan_vmoptions "../MEGAN.vmoptions" \
            -with-report MBARC_report.html \
            --queries_dir queries


# --lineage "Clostridium","Ruminiclostridium","Coraliomargarita",\
#           "Corynebacterium","Desulfosporosinus","Desulfotomaculum",\
#           "Echinicola","Escherichia","Fervidobacterium","Frateuria",\
#           "Halovivax","Hirschia","Meiothermus","Natronobacterium",\
#           "Natronococcus","Nocardiopsis","Olsenella","Pseudomonas",\
#           "Salmonella","Segniliparus","Sediminispirochaeta","Streptococcus",\
#           "Terriglobus","Thermobacillus","Enterobacteriaceae" \
```

GraftM

Run and time GraftM on MBARC data (full, 10% and 1% subsampled)

```
mkdir GraftM_output_1perc
/usr/bin/time --output=graftM_1perc_timed.txt -v bash MBARC_GraftM.sh
    "MBARC/fastq/SRR3656745.1perc.fastq.gz" "GraftM_output_1perc"
```

```
mkdir GraftM_output_10perc
/usr/bin/time --output=graftM_10perc_timed.txt -v bash MBARC_GraftM.sh "MBARC/fastq/SRR3656745.10perc.fastq.gz" "GraftM_output_10perc"
mkdir GraftM_output_timed
/usr/bin/time --output=graftM_timed.txt -v bash MBARC_GraftM.sh "MBARC/fastq/SRR3656745.fastq.gz" "GraftM_output_timed"
```

Where `MBARC_GraftM.sh` look like this:

```
for gpkg in rp16_added_gpkg/*
do
  base=$(basename ${gpkg%%.gpkg})
  singularity exec -B $PWD:$PWD graftM.simg graftM graft \
                                               --forward $1 \
                                               --graftm_package $gpkg \
                                               --input_sequence_type nucleotide \
                                               --search_method hmmsearch \
                                               --assignment_method pplacer \
                                               --threads 10 \
                                               --verbosity 5 \
                                               --log $2/"$base"_GraftM.log \
                                               --output_directory $2/"$base"
done
```

Leave one out benchmark

Prepare the leave on out benchmark as well to check how the performance is when leaving out sequences from the correct genera or family:

```
from Bio import SeqIO
from ete3 import Tree
import glob
import os
import shutil
from collections import defaultdict
import subprocess

MBARC_taxa = {'Clostridium':'Clostridiaceae',
    'Ruminiclostridium':'Ruminococcaceae',
    'Coraliomargarita':'Puniceicoccaceae',
    'Corynebacterium':'Corynebacteriaceae',
    'Desulfosporosinus':'Peptococcaceae',
    'Desulfotomaculum':'Peptococcaceae',
    'Echinicola':'Cyclobacteriaceae',
    'Escherichia':'Enterobacteriaceae',
    'Fervidobacterium':'Fervidobacteriaceae',
    'Frateuria':'Rhodanobacteraceae',
    'Halovivax':'Natrialbaceae',
    'Hirschia':'Hyphomonadaceae',
    'Olsenella':'Atopobiaceae',
    'Pseudomonas':'Pseudomonadaceae',
    'Salmonella':'Enterobacteriaceae',
    'Segniliparus':'Segniliparaceae',
```

```
    'Sediminispirochaeta':'Spirochaetaceae',
    'Meiothermus':'Thermaceae',
    'Natronobacterium':'Natrialbaceae',
    'Natronococcus':'Natrialbaceae',
    'Streptococcus':'Streptococcaceae',
    'Terriglobus':'Acidobacteriaceae',
    'Thermobacillus':'Paenibacillaceae',
    'Acidobacteriaceae':'Acidobacteriales',
    'Atopobiaceae':'Coriobacteriales',
    'Clostridiaceae':'Clostridiales',
    'Corynebacteriaceae':'Corynebacteriales',
    'Cyclobacteriaceae':'Cytophagales',
    'Enterobacteriaceae':'Enterobacterales',
    'Fervidobacteriaceae':'Thermotogales',
    'Hyphomonadaceae':'Rhodobacterales',
    'Natrialbaceae':'Natrialbales',
    'Paenibacillaceae':'Bacillales',
    'Peptococcaceae':'Clostridiales',
    'Pseudomonadaceae':'Pseudomonadales',
    'Puniceicoccaceae':'Puniceicoccales',
    'Rhodanobacteraceae':'Xanthomonadales',
    'Ruminococcaceae':'Clostridiales',
    'Segniliparaceae':'Corynebacteriales',
    'Spirochaetaceae':'Spirochaetales',
    'Streptococcaceae':'Lactobacillales',
    'Thermaceae':'Thermales'}

MBARC_loo = defaultdict(list)
for k, v in MBARC_taxa.items():
    MBARC_loo[v].append(k)

ref_dir = "../rp16_loo_references"

def check_loo_possible(base, taxa, parent):
    parent_represented = False
    sequences_removed = False
    for line in open("{}/{}/{}.taxid.map".format(ref_dir, base, base)):
        if any("{};".format(t) in line for t in taxa) or any("{}\n".format(t) in line
            for t in taxa):
            sequences_removed = True
        elif "{};".format(parent) in line or "{}\n".format(parent) in line:
            parent_represented = True
    return parent_represented and sequences_removed


def get_seqnames_prune_map(base, taxa, target_base):
    seqnames  = []
    with open("{}/{}/{}.taxid.map".format(ref_dir, target_base, target_base), 'w') as
        out:
        for line in open("{}/{}/{}.taxid.map".format(ref_dir, base, base)):
```

```python
            if any("{};".format(t) in line for t in taxa) or any("{}\n".format(t) in line for t in taxa):
                seqnames.append(line.split('\t')[0])
            else:
                print(line, file=out, end='')
    return seqnames


def prune_eggno_map(base, seqnames, target_base):
    with open("{}/{}/{}.class".format(ref_dir, target_base, target_base), 'w') as out:
        for line in open("{}/{}/{}.class".format(ref_dir, base, base)):
            print(line.replace(base, target_base), file=out, end='')
    with open("{}/{}/{}.eggnog.map".format(ref_dir, target_base, target_base), 'w') as out:
        for line in open("{}/{}/{}.eggnog.map".format(ref_dir, base, base)):
            if not line.split('\t')[0] in seqnames:
                print(line, file=out, end='')


def prune_fasta(base, seqnames, target_base, extension):
    with open("{}/{}/{}.{}".format(ref_dir, target_base, target_base, extension), 'w') as out:
        for rec in SeqIO.parse("{}/{}/{}.{}".format(ref_dir, base, base, extension), 'fasta'):
            if not rec.id in seqnames:
                SeqIO.write(rec, out, 'fasta')


def fix_alignment_all_gaps(target_base):
    cmd = "singularity exec -B $PWD PhyloMagnet.sif trimal -in {} -out {} -gt 0.0 -fasta".format(
    "{}/{}/{}.unique.aln".format(ref_dir, target_base, target_base),
    "{}/{}/{}.unique.aln".format(ref_dir, target_base, target_base))
    subprocess.call(cmd.split())


def prune_tree(base, seqnames, target_base):
    tree = Tree("{}/{}/{}.treefile".format(ref_dir, base, base), format=0)
    keep_leaves = [l.name for l in tree.iter_leaves()]
    for l in tree.iter_leaves():
        if l.name in seqnames:
            keep_leaves.remove(l.name)
    tree.prune(keep_leaves, preserve_branch_length=True)
    tree.write(outfile="{}/{}/{}.treefile".format(ref_dir, target_base, target_base), format=5)


for ref in glob.glob("{}/*_add".format(ref_dir)):
    base = os.path.basename(ref)
    for parent, taxa in MBARC_loo.items():
        if check_loo_possible(base, taxa, parent):
```

```
            target_base = base.replace('add', '{}'.format(parent))
            os.mkdir("{}/{}".format(ref_dir, target_base))

            seqnames = get_seqnames_prune_map(base, taxa, target_base)
            prune_eggno_map(base, seqnames, target_base)
            for extension in ['fasta', 'unique.aln', 'unique.fasta']:
                prune_fasta(base, seqnames, target_base, extension)
            fix_alignment_all_gaps(target_base)
            prune_tree(base, seqnames, target_base)
            shutil.copyfile("{}/{}/{}.modelfile".format(ref_dir, base, base),
                            "{}/{}/{}.modelfile".format(ref_dir, target_base,
                                target_base))
```

And run PhyloMagnet with each set of curated references:

```
import glob
import shutil
import os
import subprocess

MBARC_taxa = {'Clostridium':'Clostridiaceae',
    'Ruminiclostridium':'Ruminococcaceae',
    'Coraliomargarita':'Puniceicoccaceae',
    'Corynebacterium':'Corynebacteriaceae',
    'Desulfosporosinus':'Peptococcaceae',
    'Desulfotomaculum':'Peptococcaceae',
    'Echinicola':'Cyclobacteriaceae',
    'Escherichia':'Enterobacteriaceae',
    'Fervidobacterium':'Fervidobacteriaceae',
    'Frateuria':'Rhodanobacteraceae',
    'Halovivax':'Natrialbaceae',
    'Hirschia':'Hyphomonadaceae',
    'Olsenella':'Atopobiaceae',
    'Pseudomonas':'Pseudomonadaceae',
    'Salmonella':'Enterobacteriaceae',
    'Segniliparus':'Segniliparaceae',
    'Sediminispirochaeta':'Spirochaetaceae',
    'Meiothermus':'Thermaceae',
    'Natronobacterium':'Natrialbaceae',
    'Natronococcus':'Natrialbaceae',
    'Streptococcus':'Streptococcaceae',
    'Terriglobus':'Acidobacteriaceae',
    'Thermobacillus':'Paenibacillaceae',
    'Acidobacteriaceae':'Acidobacteriales',
    'Atopobiaceae':'Coriobacteriales',
    'Clostridiaceae':'Clostridiales',
    'Corynebacteriaceae':'Corynebacteriales',
    'Cyclobacteriaceae':'Cytophagales',
    'Enterobacteriaceae':'Enterobacterales',
    'Fervidobacteriaceae':'Thermotogales',
    'Hyphomonadaceae':'Rhodobacterales',
    'Natrialbaceae':'Natrialbales',
```

```
    'Paenibacillaceae':'Bacillales',
    'Peptococcaceae':'Clostridiales',
    'Pseudomonadaceae':'Pseudomonadales',
    'Puniceicoccaceae':'Puniceicoccales',
    'Rhodanobacteraceae':'Xanthomonadales',
    'Ruminococcaceae':'Clostridiales',
    'Segniliparaceae':'Corynebacteriales',
    'Spirochaetaceae':'Spirochaetales',
    'Streptococcaceae':'Lactobacillales',
    'Thermaceae':'Thermales'}
    # 'Nocardiopsaceae':'Streptosporangiales',
    # 'Nocardiopsis':'Nocardiopsaceae',

def run_PhyloMagnet(taxon):
    cmd = """
mkdir -p {}
cd {}
nextflow run ../PhyloMagnet/main.nf \
--lineage 'family' \
--reference_packages '../rp16_loo_rpkg/*{}.tgz' \
--fastq /proj/uppstore2017169/private/PhyloMagnet/SRR3656745.fastq.gz \
--megan_vmoptions ../MEGAN.vmoptions \
--queries_dir queries_{} \
--project snic2019-3-28 \
-with-singularity ../PhyloMagnet.sif \
-profile slurm \
-resume 2> {}.err > {}.log &
cd ..
sleep 2m
    """.format(taxon, taxon, taxon, taxon, taxon, taxon, taxon, taxon)
    print(cmd)


for taxon in set(MBARC_taxa.values()):
    if glob.glob("rp16_loo_rpkg/*{}.tgz".format(taxon)):
        run_PhyloMagnet(taxon)
```

Kraken2

Finally run Kraken2 on the MBARC dataset to compare its performance:

```
cd kraken
sudo singularity build kraken.sif Singularity

singularity exec -B $PWD kraken.sif kraken2-build --standard --protein --db standard
    --threads 10

/usr/bin/time --output=kraken_standard_time.txt -v singularity exec \
      -B $PWD kraken.sif kraken2 --db standard --gzip-compressed \
      --use-names ../MBARC/fastq/SRR3656745.fastq.gz --threads 10 \
      --output SRR3656745.out --report SRR3656745.report
cd ..
```

Tara Southern Oceans Benchmark

Run PhyloMagnet on the Coral Bleaching metatranscriptomic dataset

```
nextflow run maxemil/PhyloMagnet \
            --lineage "family" \
            -with-singularity  ../PhyloMagnet.simg \
            --cpus 32 \
            --fastq "fastq/*.fastq.gz" \
            --reference_packages "../rp16_rpkg/*" \
            --megan_vmoptions "../MEGAN.vmoptions"
```

Coral Bleaching Benchmark

Run PhyloMagnet on the Tara Oceans metagenomic dataset

```
nextflow run maxemil/PhyloMagnet \
            --lineage "family","Dinophyceae" \
            -with-singularity  ../PhyloMagnet.simg \
            --cpus 40 \
            --reference_packages "../chloroplast_rpkgs/*" \
            --megan_vmoptions "../MEGAN.vmoptions" \
            --fastq "fastq/PRJNA377366.fastq.gz" \
            -with-report "Coral_Bleaching.html"
```

Evaluate Benchmarks

Fig 2:

```
import matplotlib
matplotlib.use('Agg')
import pandas as pd
import seaborn as sns
from itertools import product
from collections import defaultdict
import matplotlib.pyplot as plt
import glob
from ete3 import ncbi_taxonomy

MBARC_genera = ['Clostridium','Ruminiclostridium','Coraliomargarita',
    'Corynebacterium','Desulfosporosinus','Desulfotomaculum','Echinicola',
    'Escherichia','Fervidobacterium','Frateuria','Halovivax','Hirschia',
    'Olsenella','Pseudomonas','Salmonella','Segniliparus','Sediminispirochaeta',
    'Meiothermus','Natronobacterium','Natronococcus','Nocardiopsis',
    'Streptococcus','Terriglobus','Thermobacillus']

MBARC_families = ['Acidobacteriaceae', 'Atopobiaceae', 'Clostridiaceae'
    'Corynebacteriaceae', 'Cyclobacteriaceae', 'Enterobacteriaceae', 'Fervidobacteriaceae'
    'Hyphomonadaceae', 'Natrialbaceae', 'Nocardiopsaceae', 'Paenibacillaceae'
    'Peptococcaceae', 'Pseudomonadaceae', 'Puniceicoccaceae', 'Rhodanobacteraceae'
```

```
    'Ruminococcaceae', 'Segniliparaceae', 'Spirochaetaceae',
        'Streptococcaceae','Thermaceae']

def get_counts_tree_taxon(df):
    taxa = set(df["Taxon"])
    samples = set(df["Tree"])
    counts = pd.DataFrame(columns=samples, index=taxa)
    counts = counts.fillna(0)
    for t,s in product(taxa, samples):
        counts[s].loc[t] = df[(df["Tree"] == s) & (df["Taxon"] == t)].shape[0]
    return counts

def parse_graftM_results(path):
    ncbi = ncbi_taxonomy.NCBITaxa()
    lineages = defaultdict(lambda: defaultdict(int))
    rank = "genus"
    for infile in glob.glob("{}/COG*/combined_count_table.txt".format(path)):
        taxids = set()
        tree = infile.split('/')[1]
        for line in open(infile):
            lineage = [l.strip() for l in line.split('\t')[2].split(';')]
            name2tax = ncbi.get_name_translator(lineage)
            taxids |= set([taxid for l in name2tax.values() for taxid in l])
        for k,v in ncbi.get_rank(taxids).items():
                if v == rank:
                    lineages[tree][ncbi.get_taxid_translator([k])[k]] += 1
    return pd.DataFrame.from_dict(lineages).fillna(0)

def parse_PhyloMagnet_results(infile):
    df = pd.read_csv(infile, sep='\t', header=None, names=['Sample', 'Tree', 'Taxon',
        'value'], dtype=str)
    df = df[df['value'] == 'True']
    return get_counts_tree_taxon(df)

def divide_tp_and_fp(df, tool):
    df = df.apply(lambda x: x if x.name in MBARC_genera else (-1 * x), axis=1)
    df_tp = pd.DataFrame({'counts':df[df >= 0].sum(), 'tool':tool})
    df_fp = pd.DataFrame({'counts':df[df <= 0].sum(), 'tool':tool})
    return df_tp.append(df_fp)

def lighten_color(color, amount=0.5):
    import matplotlib.colors as mc
    import colorsys
    try:
        c = mc.cnames[color]
    except:
        c = color
    c = colorsys.rgb_to_hls(*mc.to_rgb(c))
    return colorsys.hls_to_rgb(c[0], 1 - amount * (1 - c[1]), c[2])

def get_counts_both_tools():
```

```
    graftm_1 = parse_graftM_results("GraftM_output_1perc")
    df = divide_tp_and_fp(graftm_1, 'GraftM 1%')
    graftm_10 = parse_graftM_results("GraftM_output_10perc")
    df = df.append(divide_tp_and_fp(graftm_10, 'GraftM 10%'))
    graftm = parse_graftM_results("GraftM_output")
    df = df.append(divide_tp_and_fp(graftm, 'GraftM full'))

    counts_1 = parse_PhyloMagnet_results("MBARC/queries_family/tree_decisions.txt")
    df = df.append(divide_tp_and_fp(counts_1, 'PhyloMagnet 1%'))
    counts_10 = parse_PhyloMagnet_results("MBARC/queries_family/tree_decisions.txt")
    df = df.append(divide_tp_and_fp(counts_10, 'PhyloMagnet 10%'))
    counts = parse_PhyloMagnet_results("MBARC/queries_family/tree_decisions.txt")
    df = df.append(divide_tp_and_fp(counts, 'PhyloMagnet full'))
    return df

def plot_counts_fp_tp(df):
    fig, ax = plt.subplots(1,1)
    # dummy plots, just to get the Path objects
    a = ax.scatter([1,2],[3,4], marker='o')
    b = ax.scatter([1,2],[3,4], marker='^')
    circle_mk, = a.get_paths()
    triangle_up_mk, = b.get_paths()
    plt.close()

    clr_palette = [lighten_color(sns.xkcd_rgb['scarlet'], 0.7),
                   lighten_color(sns.xkcd_rgb['scarlet'], 1),
                   lighten_color(sns.xkcd_rgb['scarlet'], 1.3),
                   lighten_color(sns.xkcd_rgb['denim'], 0.7),
                   lighten_color(sns.xkcd_rgb['denim'], 1),
                   lighten_color(sns.xkcd_rgb['denim'], 1.3)]
    markers = [triangle_up_mk, triangle_up_mk, triangle_up_mk, circle_mk, circle_mk,
        circle_mk]
    clr2mrk = {int(c[0]*1000):m for c,m in zip(clr_palette, markers)}

    fig, ax = plt.subplots(figsize=(10,6), tight_layout=True)
    sns.swarmplot(x=df.index, y='counts', data=df, hue='tool',
                   ax=ax, palette=clr_palette, linewidth=0.4, size=6)
    # i = 0
    # for c in ax.collections:
    #     if len(c.get_facecolors()) == 12:
    #         paths = []
    #         for f in c.get_facecolors():
    #             paths.append(clr2mrk[int(f[0]*1000)])
    #         c.set_paths(paths)
    #     else:
    #         c.set_paths([paths[i]])
    #         i += 1

    ax.set_xticklabels(labels=df.index, rotation=90)
    ax.set_ylabel('True and False positive')
    ax.set_ylim(-16, 25)
```

```
    ax.axhline(0, color=sns.xkcd_rgb['denim'])
    ax.legend(frameon=False, bbox_to_anchor=(1, 1), loc=2)
    fig.savefig('Fig2_genus.pdf', orientation='landscape', dpi=500)

if __name__ == '__main__':
    df = get_counts_both_tools()
    plot_counts_fp_tp(df)
```

Fig 3:

This figure is simply the heatmap taken from the ouput of the Tara oceans benchmark, added with a 'taxonomic tree' as it can be found on ncbi. `Tara_Southern_Ocean/queries/decision_heatmap.pdf`

Fig 4:

These are the two trees `Coral_Bleaching/queries/PRJNA377366/PRJNA377366-psbb.newick` and `Coral_Bleaching/place_transcripts/psbb.newick` aligned to each other so as to be able to compare the placements

Fig S1:

```
import matplotlib
matplotlib.use('Agg')
import pandas as pd
import seaborn as sns
from itertools import product
from collections import defaultdict
import matplotlib.pyplot as plt
import glob
from ete3 import ncbi_taxonomy

def get_counts_sample_taxon(df):
    taxa = set(df["Taxon"])
    samples = set(df["Sample"])
    counts = pd.DataFrame(columns=samples, index=taxa)
    counts = counts.fillna(0)
    for t,s in product(taxa, samples):
        counts[s].loc[t] = df[(df["Sample"] == s) & (df["Taxon"] == t)].shape[0]
    return counts

def parse_graftM_results_sample(path):
    ncbi = ncbi_taxonomy.NCBITaxa()
    lineages = defaultdict(int)
    rank = "genus"
    for infile in glob.glob("{}/COG*/combined_count_table.txt".format(path)):
        taxids = set()
        for line in open(infile):
            lineage = [l.strip() for l in line.split('\t')[2].split(';')]
            name2tax = ncbi.get_name_translator(lineage)
            taxids |= set([taxid for l in name2tax.values() for taxid in l])
        for k,v in ncbi.get_rank(taxids).items():
                if v == rank:
```

```
                    lineages[ncbi.get_taxid_translator([k])[k]] += 1
    return pd.DataFrame.from_dict(lineages,orient='index')

def parse_PhyloMagnet_results_sample(infile):
    df = pd.read_csv(infile, sep='\t', header=None, names=['Sample', 'Tree', 'Taxon',
        'value'], dtype=str)
    df = df[df['value'] == 'True']
    return get_counts_sample_taxon(df)

def get_counts_both_tools_sample():
    phylomagnet = parse_PhyloMagnet_results_sample("MBARC/queries/tree_decisions.txt")
    graftm = parse_graftM_results_sample("GraftM_output")
    return (phylomagnet, graftm)

def plot_heatmap(phylomagnet, graftm):
    fig, ax = plt.subplots(figsize=(10,20), tight_layout=True)
    compare = pd.DataFrame({'GraftM':graftm[0],'PhyloMagnet':phylomagnet.SRR3656745})
    compare = compare.fillna(0)
    compare = compare.sort_values(by=['PhyloMagnet', 'GraftM'],ascending=False)
    sns.heatmap(compare, annot=True, cmap='Reds', xticklabels=True, ax=ax)
    fig.savefig('FigS1.pdf')

if __name__ == '__main__':
    phylomagnet, graftm = get_counts_both_tools_sample()
    plot_heatmap(phylomagnet, graftm)
```

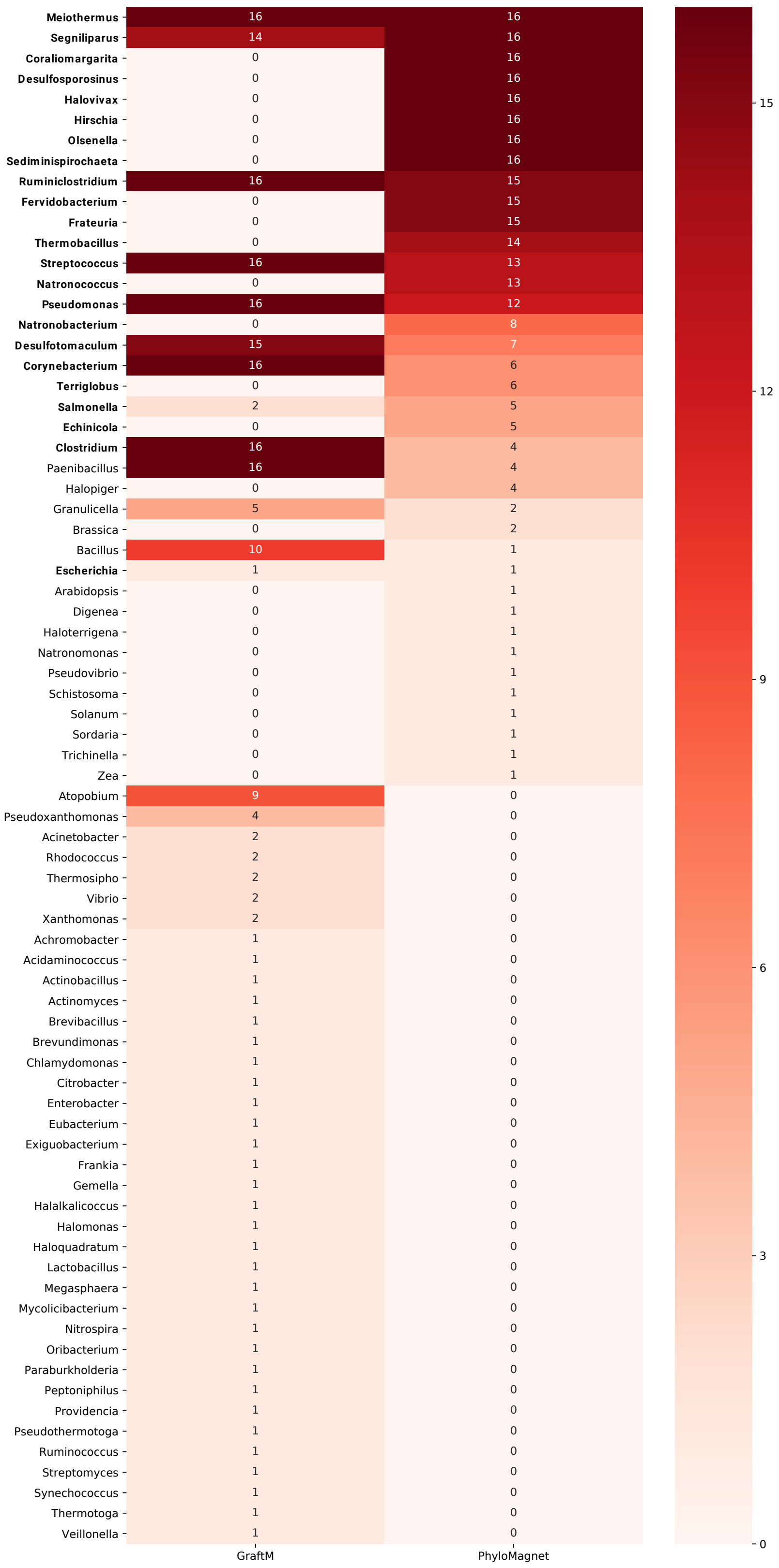

Figure S1: Classification results of PhyloMagnet and GraftM on the full MBARC-26 dataset. Values and colors correspond to the number of rp16 trees a genus was identified in. Those genera that are part of the MBARC-26 community are written in bold.

Fig S2:

```python
import matplotlib.pyplot as plt
import pandas as pd
import seaborn as sns


fig, ax = plt.subplots(2, 1, figsize=(20,10), tight_layout=True)

df = pd.DataFrame.from_csv('MBARC/runtimes.csv',index_col=None)
sns.catplot(x='size', y='time',hue='tool', data=df, ax=ax[0], kind="bar",
    palette="muted", legend=False)
ax[0].set(xlabel="size [Gb]", ylabel='time [s]')

df = pd.DataFrame.from_csv('MBARC/memory.csv',index_col=None)
sns.catplot(x='size', y='memory',hue='tool', data=df, ax=ax[1], kind="bar",
    palette="muted", legend_out=True)
ax[1].set(xlabel="size [Gb]", ylabel='memory [GB]')

fig.savefig('FigS2.pdf')
```

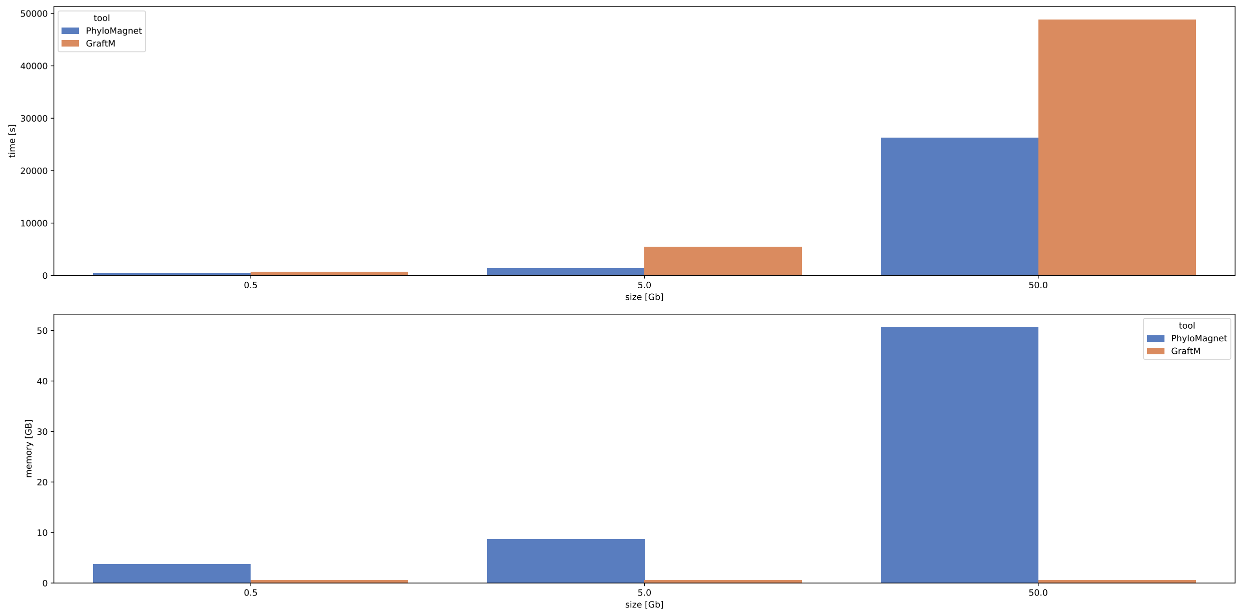

Figure S2: Computational footprint of PhyloMagnet and GraftM for the complete MBARC-26 dataset (50Gb), the subsample of 1% (5Gb) and of 10% (0.5Gb). top: runtime on 10 CPUs. bottom: peak memory usage.

Fig S3:

```python
import seaborn as sns
import pandas as pd
import glob
import matplotlib.pyplot as plt

```

```
df = None
for d in glob.glob("MBARC/loo_results/*"):
    trues = 0.0
    totals = 0.0
    for line in open("{}/queries_{}/tree_decisions.txt".format(d, d)):
        line = line.strip().split('\t')
        if line[2] == d:
            totals +=1
            if line[3] == "True":
                trues +=1
    if "aceae" in d:
        level = 'Family'
    elif "ales" in d:
        level = 'Order'
    p_df = pd.DataFrame({"frac":(trues/totals)*100, "level":level}, index=[d])
    if df is None:
        df = p_df
    else:
        df = df.append(p_df)


fig, ax = plt.subplots(figsize=(10,6), tight_layout=True)

sns.violinplot(x="level", y="frac", data=df, inner="box", cut=0, ax=ax)
# ax = sns.boxplot(x="level", y="frac", data=df)
ax.set_ylabel("fraction of recoverd proteins [%]")
ax.set_xlabel("")
fig.savefig('FigS3.pdf', orientation='landscape', dpi=500)
```

Fig S4:

```
import seaborn as sns
import pandas as pd
import matplotlib.pyplot as plt

MBARC_genera = ['Clostridium','Ruminiclostridium','Coraliomargarita',
    'Corynebacterium','Desulfosporosinus','Desulfotomaculum','Echinicola',
    'Escherichia','Fervidobacterium','Frateuria','Halovivax','Hirschia',
    'Olsenella','Pseudomonas','Salmonella','Segniliparus','Sediminispirochaeta',
    'Meiothermus','Natronobacterium','Natronococcus','Nocardiopsis',
    'Streptococcus','Terriglobus','Thermobacillus']

df = pd.read_csv("kraken/SRR3656745.report", sep="\t", header=None)

df = df[df[3] == "G"]
df = df.sort_values(by=1, ascending=True)
df = df[df[1] > 1000]

colors = list(df.apply(lambda x: 'red' if any([x[5].lstrip() == t for t in
    MBARC_genera]) else 'black', axis=1))#

fig = plt.figure()
```

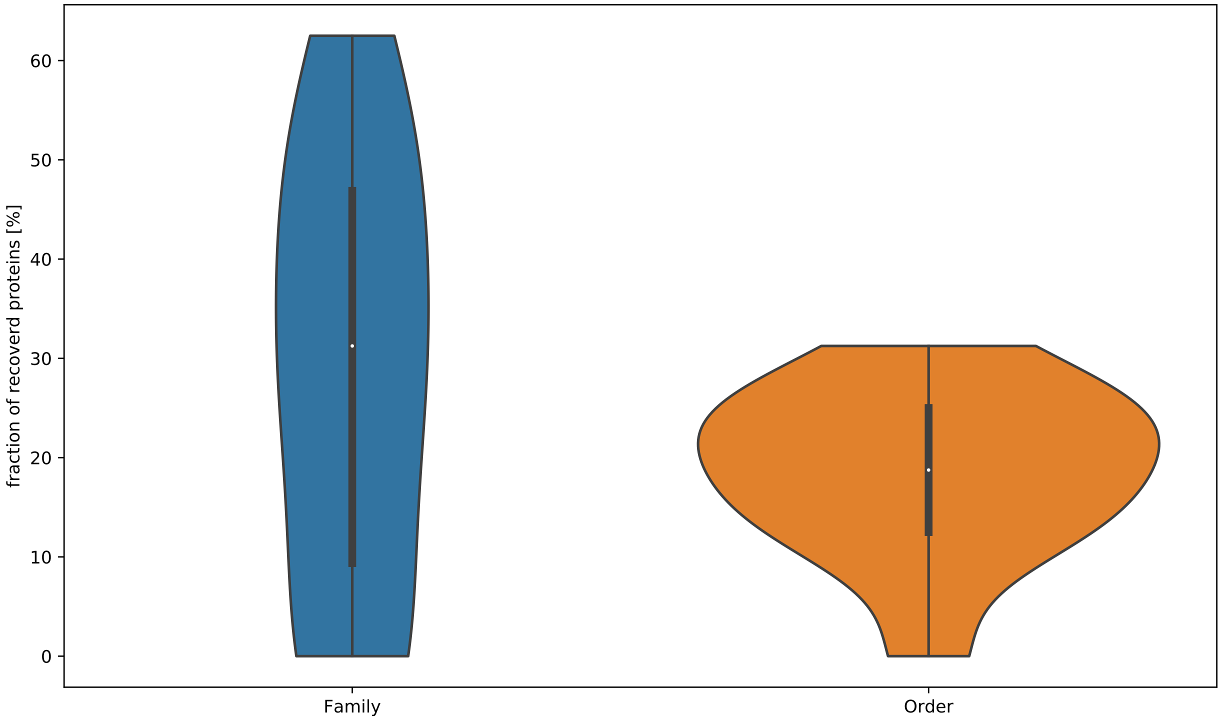

Figure S3: Violon- and boxplots showing the performance of PhyloMagnet on the MBARC-26 dataset at family and order level if the correct genus or family, respectively, was excluded.

```
ax = df[1].plot(kind="barh", color=colors, logx=True, figsize=(7,20))
ax.set_xlabel("no. of assigned reads")
ax.set_ylabel("Genera sorted by no. of assigned reads")
ax.set_yticklabels([])
ax.set_yticks([])
plt.plot(0, 0, color='black')
plt.plot(0, 0, color='red')
ax.legend(["non-MBARC genera", 'MBARC genera'])
fig.savefig('FigS4.pdf')
```

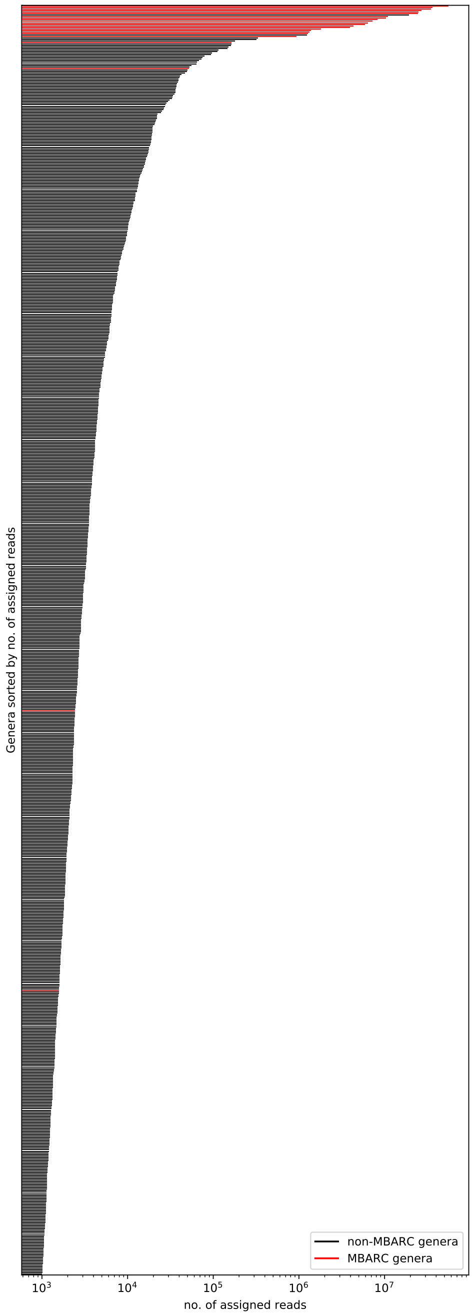

Figure S4: Barplot showing the classifiction of reads by Kraken2 at genus level. Genera that are part of MBARC-26 are highlighted in red, others are black. The y-axis is sorted by number of reads (and only genera with a more than 1000 assigned reads are shown). The x-axis shows the number of reads assigned to a genus and is logarithmic.

Table S1:

Table S1: Organisms included in the MBARC-26 dataset. For each organism, current taxonomy (species, genus and family as given in the ncbi taxonomy), ncbi assembly ID and percentage mapped illumina reads as presented in Singer *et al.* (2018) are shown.

genus	species	family	Assembly	TaxID	mapped reads [%]
Desulfosporosinus	acidiphilus	Peptococcaceae	NC_018068	646529	15.11
Sediminispirochaeta	smaragdinae	Spirochaetaceae	NC_014364	573413	11.39
Fervidobacterium	pennivorans	Fervidobacteriaceae	NC_017095	771875	11.26
Meiothermus	Silvanus	Thermaceae	NC_014212	526227	8.56
Thermobacillus	composti	Paenibacillaceae	NC_019897	717605	8.5
Hirschia	baltica	Hyphomonadaceae	NC_012982	582402	8.16
Desulfotomaculum	gibsoniae	Peptococcaceae	NC_021184	767817	6.91
Desulfosporosinus	meridiei	Peptococcaceae	NC_018515	768704	4.61
Frateuria	aurantia	Rhodanobacteraceae	NC_017033	767434	3.99
Natronococcus	occultus	Natrialbaceae	NC_019974.1	694430	3.55
Coraliomargarita	akajimensis	Puniceicoccaceae	NC_014008	583355	3.41
Natronobacterium	gregoryi	Natrialbaceae	NC_019792.1	797304	2.46
Olsenella	uli	Atopobiaceae	NC_014363	633147	2.26
Terriglobus	roseus	Acidobacteriaceae	NC_018014	926566	2.07
Halovivax	ruber	Natrialbaceae	CP003050.1	797302	1.75
Pseudomonas	stutzeri	Pseudomonadaceae	NC_019936	644801	1.55
Segniliparus	rotundus	Segniliparaceae	NC_014168	640132	1.41
Echinicola	vietnamensis	Cyclobacteriaceae	NC_019904	926556	0.62
Salmonella	enterica	Enterobacteriaceae	NC_010067	41514	0.52
Ruminiclostridium	thermocellum	Ruminococcaceae	NC_009012	203119	0.43
Streptococcus	pyogenes	Streptococcaceae	NC_002737	160490	0.43
Clostridium	perfringens	Clostridiaceae	NC_008261	195103	0.42
Corynebacterium	glutamicum	Corynebacteriaceae	NC_003450	196627	0.3
Escherichia	coli	Enterobacteriaceae	NC_000913	511145	0.18
Salmonella	bongori	Enterobacteriaceae	NC_015761	218493	0.14
Nocardiopsis	dassonvillei	Nocardiopsaceae	NC_014211	446468	0

Table S2:

Table S2: Taxonomic annotation of MAGs extracted from the Tara southern oceans dataset by Delmont *et. al* (2018). The inferred taxonomic labels from domain to species level (as far as available) are provided for the 11 prokaryotic nonredundant MAGs (inferred with GraftM; annotation of TARA_ANE_MAG_00007 is given for TARA_SOC_MAG_00009, as they have ANI >99%) as well as for those additional raw bins where a taxonomic annotation could be inferred with sourmash.

MAG/Bin	Phylum	Class	Order	Family
TARA_SOC_MAG_00001	Proteobacteria	Alphaproteobacteria	-	-
TARA_SOC_MAG_00002	Proteobacteria	Gammaproteobacteria	Alteromonadales	Alteromonadaceae
TARA_SOC_MAG_00003	Bacteroidetes	Flavobacteriia	Flavobacteriales	-
TARA_SOC_MAG_00004	Bacteroidetes	Flavobacteriia	Flavobacteriales	-
TARA_SOC_MAG_00005	Bacteroidetes	Flavobacteriia	Flavobacteriales	-
TARA_SOC_MAG_00006	Bacteroidetes	Flavobacteriia	Flavobacteriales	**Cryomorphaceae**
TARA_SOC_MAG_00007	Proteobacteria	Gammaproteobacteria	-	-
TARA_SOC_MAG_00008	Proteobacteria	Alphaproteobacteria	Rickettsiales	-
TARA_SOC_MAG_00009	Actinobacteria	Actinobacteria	Actinomycetales	Microbacteriaceae
TARA_SOC_MAG_00010	Proteobacteria	Gammaproteobacteria	-	-
TARA_SOC_MAG_00011	Bacteroidetes	Flavobacteriia	Flavobacteriales	Flavobacteriaceae
TARA_SOC_MAG_00012	Bacteroidetes	Flavobacteriia	Flavobacteriales	-
TARA_SOC_Bin_00046	Bacteroidetes	Flavobacteriia	Flavobacteriales	Flavobacteriaceae
TARA_SOC_Bin_00048	Proteobacteria	Alphaproteobacteria	Pelagibacterales	Pelagibacteraceae
TARA_SOC_Bin_00060	Bacteroidetes	Flavobacteriia	Flavobacteriales	Flavobacteriaceae
TARA_SOC_Bin_00063	Proteobacteria	Gammaproteobacteria	Cellvibrionales	Porticoccaceae
TARA_SOC_Bin_00074	Proteobacteria	Alphaproteobacteria	Pelagibacterales	Pelagibacteraceae
TARA_SOC_Bin_00075	Proteobacteria	Gammaproteobacteria	Cellvibrionales	Porticoccaceae
TARA_SOC_Bin_00095	Bacteroidetes	Flavobacteriia	Flavobacteriales	Cryomorphaceae
TARA_SOC_Bin_00115	Proteobacteria	Gammaproteobacteria	Cellvibrionales	Porticoccaceae
TARA_SOC_Bin_00226	Proteobacteria	Gammaproteobacteria	Cellvibrionales	Porticoccaceae
TARA_SOC_Bin_00242	Proteobacteria	Alphaproteobacteria	Rhodobacterales	Rhodobacteraceae

Table S2: continued

MAG/Bin	Genus	Species	Tool
TARA_SOC_MAG_00001	-	-	GraftM
TARA_SOC_MAG_00002	-	-	GraftM
TARA_SOC_MAG_00003	-	-	GraftM
TARA_SOC_MAG_00004	-	-	GraftM
TARA_SOC_MAG_00005	-	-	GraftM
TARA_SOC_MAG_00006	**unassigned**	**Cryomorphaceae bacterium ASP10-05a**	GraftM/**sourmash**
TARA_SOC_MAG_00007	-	-	GraftM
TARA_SOC_MAG_00008	-	-	GraftM
TARA_SOC_MAG_00009	Microbacterium	-	GraftM
TARA_SOC_MAG_00010	-	-	GraftM
TARA_SOC_MAG_00011	Polaribacter	-	GraftM
TARA_SOC_MAG_00012	-	-	GraftM
TARA_SOC_Bin_00046	-	Flavobacteriaceae bacterium ASP10-09a	sourmash
TARA_SOC_Bin_00048	Candidatus Pelagibacter	Candidatus Pelagibacter sp. IMCC9063	sourmash
TARA_SOC_Bin_00060	Flavobacterium	Flavobacterium sp. SCGC AAA160-P02	sourmash
TARA_SOC_Bin_00063	-	marine gamma proteobacterium ASP10-03a	sourmash
TARA_SOC_Bin_00074	Candidatus Pelagibacter	Candidatus Pelagibacter sp. IMCC9063	sourmash
TARA_SOC_Bin_00075	-	marine gamma proteobacterium ASP10-03a	sourmash
TARA_SOC_Bin_00095	-	Cryomorphaceae bacterium ASP10-05a	sourmash
TARA_SOC_Bin_00115	-	marine gamma proteobacterium ASP10-03a	sourmash
TARA_SOC_Bin_00226	-	marine gamma proteobacterium ASP10-03a	sourmash
TARA_SOC_Bin_00242	-	Rhodobacteraceae bacterium ASP10-04a	sourmash

References

Altschul, S. F., Madden, T. L., Schäffer, A. A., Zhang, J., Zhang, Z., Miller, W., & Lipman, D. J. (1997). Gapped BLAST and PSI-BLAST: a new generation of protein database search programs. Nucleic Acids Research, 25(17), 3389–402. Retrieved from http://www.ncbi.nlm.nih.gov/pubmed/9254694

Boyd, J. A., Woodcroft, B. J., & Tyson, G. W. (2018). GraftM: a tool for scalable, phylogenetically informed classification of genes within metagenomes. Nucleic Acids Research, 46(10), e59–e59. https://doi.org/10.1093/nar/gky174

Di Tommaso, P., Chatzou, M., Floden, E. W., Barja, P. P., Palumbo, E., & Notredame, C. (2017). Nextflow enables reproducible computational workflows. Nature Biotechnology, 35(4), 316–319. https://doi.org/10.1038/nbt.3820

Eddy, S. R. (2011). Accelerated Profile HMM Searches. PLoS Computational Biology, 7(10), e1002195. https://doi.org/10.1371/journal.pcbi.1002195

Katoh, K., & Standley, D. M. (2013). MAFFT multiple sequence alignment software version 7: Improvements in performance and usability. Molecular Biology and Evolution, 30(4), 772–780. https://doi.org/10.1093/molbev/mst010

Nguyen, L. T., Schmidt, H. A., Von Haeseler, A., & Minh, B. Q. (2015). IQ-TREE: A fast and effective stochastic algorithm for estimating maximum-likelihood phylogenies. Molecular Biology and Evolution, 32(1), 268–274. https://doi.org/10.1093/molbev/msu300

Titus Brown, C., & Irber, L. (2016). sourmash: a library for MinHash sketching of DNA. The Journal of Open Source Software, 1(5), 27. https://doi.org/10.21105/joss.00027
